# Alas, Poor Summers!

**Published:** 1899-08

**Authors:** 


### TEXAS MEDICAL JOURNAL.

AUSTIN, TEXAS.

A MONTHLY JOURNAL OF MEDICINE AND SURGERY.

F. E. DANIEL, M. D., Editor.

Published Monthly at Austin, Texas, by Drs. Daniel and Hudson. Subscription price $1.00 a year in advance.

Eastern Representative: John Guy Monihan, St. Paul Building, 220 Broadway, New York City.

Official organ of the West Texas Medical Association, the Houston District Medical Association, the Austin District Medical Society, the Brazos Valley Medical Association, the Galveston County Medical Society, and several others.

### Alas, Poor Summers!

Dr. Thomas Osmond Summers suicided in St. Louis, June 19th.

"The man who gets up
A filled guest from the banquet and drains off his cup,
Sees the last light extinguished, with cheerfulness goes
Well contented to bed and enjoys his repose;
But he who hath seen the wine flow, by himself scarce tasted,
Heard the music but missed the tune, and who hath wasted
A part of life's grand possibilities,—friend,
That man will bear with him, be sure, to the end,
A blighted existence and a rancor within."

Dr. Summers was unfitted by nature for the rough and tumble fight of life—the scuffle for existence—the pursuit of the almighty dollar; he couldn't "rustle." His sensitive nature shrank from the methods now in vogue and which alone seem successful in capturing the "filthy lucre;" he regarded it still as "filthy lucre," but recognized that it is the one thing needful; he says he "lost life's fight." Had he been a man of wealth, instead of having to struggle for existence, his brilliant qualities would have won for him distinction; as a poet he would have won a place in the temple of fame. Like Poe and Chatterton, he was "unfit" physically and mentally for the struggle; but like them, too, let us hope that he will live in the memory of man by the few gems of poetic inspiration he has left; that man will accord to him now that appreciation which he felt was withheld from him in life. His last poem was like the "song of the dying swan," and is the best that he has left us. We reproduce it from the *Medical Fortnightly*. The poem, and the letter to his wife, which was found on his body, tell the story of his life. He had, in envy, perhaps, seen the wine flow,—at a distance; had heard the music,—at a distance,—and had missed the tune; he had wasted a part,—perhaps a large part,—of life's grand possibilities, and his was, in his mind, a blighted existence; he had lost life's fight, and so dropped out of the ranks.

PERDI VITAM VALE MUNDUM.

"Good night, old world, good-by to all your joys,
Your sorrows, pleasures, passions, pomps and noise.
I leave you for th' eternal silence of the stars—
The deafness of unbounded space, where bars
No longer hold the soul in durance vile—
Where naught can wound and nothing can defile.
There the pure spirit shall despise the things
The sense on earth hath loved. On wings
Bathed in the ether of eternity—
How sweet to feel from every passion free!
And yet—it is an awful leap to take
Into the great unknown! perchance to wake
To greater woes, indeed, than those we have
And hoped to bury in the silent grave.
But still, the great majority is there!
Or tremble when the hour supreme has come,
As soon or late it must? Man's final home,
The grave, at least gives rest from troubles here,
And we may hope for sweet oblivion there.
Then, Charon, come! I signal thee tonight!
Come row me o'er the Styx—I've lost life's fight!"

The letter to Mrs. Summers was written in lead pencil. It read:

"June 18, 1899.

"My Darling Wife: I have reached that point when Azonel's thread is all that is left to me. I feel well assured that your love and esteem will crown my memory when they could not bless my life. I die that you may live. Think of me as I was in the palmier days of life, and let the mantle of your charity cover those faults which were more infirmities than sins, engendered in despondency and cultivated by disappointment. I send the bullet through my brain instead of my heart, that you may know its last throb beat for you and the dear ones I leave with such ineffable sorrow. Good by. OSMOND."

The *Medical Fortnightly* says: "No one who ever knew Dr. Summers will ever give his memory anything but sympathetic, compassionate reverence. He suffered much. In his 'palmier days' he was one of nature's noblemen; later, he was practically an invalid, and when well nigh crushed by disappointment, and what seemed to his over-sensitive nature, ingratitude, he committed the final act which cannot be undone. How many friends he really had he may not know. Would that all might know what he suffered, and might have helped make life for him worth living."

---

THE REPORT of the health officer of San Antonio shows that of the 1440 deaths for the year ending June 30, 1899, 315 were from consumption; 174 amongst non-residents, 131 residents longer than one year. Dr. Paschall, the health officer, has instituted sanitary reforms which will greatly lower this enormous death rate. San Antonio has a half million dollar sewer system, but it seems difficult to make everybody connect with it, and to abolish the old privy vaults and surface deposit abominations. He urges upon the city authorities the necessity of passing stringent ordinances on this subject,—a house to house inspection, inspection of house plumbing and other details of a strict sanitary system. The slogan note of "Sanitary Reform" has been caught by San Antonio and Houston, and there is a shaking up of dry bones. We live yet in hopes that it may strike our lawmakers and our Governor,—but it is slow work "educating the masses"—and "classes."

---

MR. E. A. HYDE, under the auspices of the Master Plumbers' League (ten Southern States), is lecturing through the South (is now at Austin) on the prevention of disease in general, and in especial, by improved plumbing. In our next issue we will have something to say on this subject. It is not generally known that the Twenty-fifth Legislature passed a bill, gotten up by the plumbers, regulating plumbing, creating a board of examiners to examine all plumbers, and issue a license to those who pass. It is the only law of the kind in existence in the United States.

THE SHOEMAKER BEYOND HIS LAST.—Dr. Flavel B. Tiffany of Kansas City, visited San Antonio during the session of the Texas State Medical Association in April last, and contributed to the Kansas City *Medical Index-Lancet* an account of his trip. Amongst other interesting things, he mentions that "The St. Alamo stands near the Menger hotel;" and that the old Spanish Catholic Missions a few miles from the city, "served as fortifications in the war with the Mexicans under General Jackson."

---

DR. DOTY, Port Health Officer at New York, has prepared a serum—from a culture made from the blood of a yellow fever patient—presumably containing the specific microbe of that disease, and has sent a lot of it to Vera Cruz to be tried. The doctor has not tried it himself on anything but guinea pigs and rabbits, but is confident it will prevent—and cure—yellow fever—and wants some other fellow to try it on the human subject.

---

W. R. WARNER & Co., Philadelphia, will send free on request, to any of the JOURNAL readers, a neatly arranged "Dose Table" for quick reference as to doses of all principal remedies now in use. The doses are given in the metric system, with their equivalent in grains, drachms, etc.

---

STILL THEY COME.—The latest "cure for consumption" is "telegraphed from France to the State Department at Washington." One, Mendel, says that oil of eucalyptus and oil of cinnamon in olive oil, *injected into the bronchial tubes*, will cure consumption. Bosh!

---

THE OUTBREAK of yellow fever at Hampton, Va., has about been "stamped out" by the M. H. S. Score for Wyman!

